# High-Density Genomewide Linkage Analysis of Exceptional Human Longevity Identifies Multiple Novel Loci

**DOI:** 10.1371/journal.pone.0012432

**Published:** 2010-08-31

**Authors:** Steven E. Boyden, Louis M. Kunkel

**Affiliations:** 1 Department of Genetics, Harvard Medical School, Boston, Massachusetts, United States of America; 2 Program in Genomics, Division of Genetics, and The Manton Center for Orphan Disease Research, Children's Hospital Boston, Boston, Massachusetts, United States of America; 3 Howard Hughes Medical Institute, Children's Hospital Boston, Boston, Massachusetts, United States of America; Ohio State University Medical Center, United States of America

## Abstract

**Background:**

Human lifespan is approximately 25% heritable, and genetic factors may be particularly important for achieving exceptional longevity. Accordingly, siblings of centenarians have a dramatically higher probability of reaching extreme old age than the general population.

**Methodology/Principal Findings:**

To map the loci conferring a survival advantage, we performed the second genomewide linkage scan on human longevity and the first using a high-density marker panel of single nucleotide polymorphisms. By systematically testing a range of minimum age cutoffs in 279 families with multiple long-lived siblings, we identified a locus on chromosome 3p24-22 with a genomewide significant allele-sharing LOD score of 4.02 (empirical *P* = 0.037) and a locus on chromosome 9q31-34 with a highly suggestive LOD score of 3.89 (empirical *P* = 0.054). The empirical *P* value for the combined result was 0.002. A third novel locus with a LOD score of 4.05 on chromosome 12q24 was detected in a subset of the data, and we also obtained modest evidence for a previously reported interval on chromosome 4q22-25.

**Conclusions/Significance:**

Our linkage data should facilitate the discovery of both common and rare variants that determine genetic variability in lifespan.

## Introduction

Many common diseases of adulthood increase in prevalence with age. These morbidities accompany an exponential increase in mortality rate that is maintained until approximately age 90, whereupon it starts to decelerate [1]. The reduction in observed versus expected mortality may be due to demographic selection, whereby individuals with alleles predisposing them to an early or average age of death, once deceased, leave behind a robust cohort depleted of detrimental alleles and/or enriched for alleles that promote longevity [2], [3]. Centenarians often reach old age with delayed onset or absence of geriatric diseases [4], possibly benefitting from a “compression of morbidity” that confines these diseases to a short duration at the end of their life [5]. This correlation between exceptional longevity and healthy aging suggests that common genetic factors may underlie both traits. The epidemiology and phenotypes characteristic of human aging and results of candidate gene association studies have been reviewed elsewhere [6]–[8]. While many variants have demonstrated preliminary evidence of association to exceptional longevity [7], the only confirmed associations are those of the *APOE* (MIM 107741) haplotypes [9]–[12]. Multiple recently reported associations between variants in *FOXO3* (MIM 602681) and longevity are also quite promising [13]–[18]. Despite these important discoveries, additional alleles that may regulate aging in humans and allow a minority of the population to attain extreme old age have likely yet to be identified.

Estimates of the heritability of normal human lifespan range from 10% to 58%, averaging about 25% [19]. The genetic contribution to lifespan grows markedly after age 60, indicating the heritability of exceptional longevity may be substantially higher than these estimates [20]. The relative survival probability for siblings of centenarians increases steadily with age, until male and female siblings have a 17-fold and 8-fold increased chance, respectively, of reaching age 100 compared to others from their birth cohort [21]. Moreover, while natural lifespan is likely a complex trait controlled by many genes with small effect sizes, extreme longevity may be determined by fewer genes of stronger effect [22], [23], and may therefore be amenable to linkage analysis. The only previous genomewide scan for linkage to longevity was conducted in part by a member of our group (LMK) and identified a region on chromosome 4q22-25 as significantly linked in 137 sibships of centenarians and nonagenarians [24]. A subsequent genomewide scan for healthy aging in a smaller and younger cohort provided weak support for the chromosome 4q22-25 linkage [25], whereas a targeted study of 164 sibships of nonagenarians did not find linkage to the locus [26], nor did a genomewide scan on bone characteristics as a biomarker for biological aging [27]. All these studies used microsatellite markers with 5–10 cM spacing. To assess the linkage to chromosome 4 and identify new loci, we performed the most powerful linkage scan to date on exceptional longevity. Though the evidence for linkage to chromosome 4 remains equivocal, several novel loci were discovered in our scan, including a region on chromosome 3p24-22 with an empirically genomewide significant LOD score of 4.02 and a region on chromosome 9q31-34 with a LOD score of 3.89.

## Methods

### Ethics Statement

Subjects were recruited through Elixir Pharmaceuticals, the New England Centenarian Study (NECS) now of Boston University Medical Center, Beth Israel Deaconess Medical Center (BIDMC), and Children's Hospital Boston (CHB), as described previously [24], [28]. All participants provided written informed consent and the study was approved by the Institutional Review Boards of the above institutions. All samples were de-identified and were either available from BIDMC or CHB, or were purchased from Elixir Pharmaceuticals or NECS for a processing fee.

### Subjects

All subjects provided proof of age. There was a predominance of female subjects in our cohort, likely reflecting the original ascertainment criterion of having a proband of at least 98 years of age regardless of gender [24]. Only self-identified white or Caucasian subjects (the vast majority of our cohort) were analyzed, since population stratification can confound nonparametric linkage analysis when parental genotypes are unobserved [29]. We had available gender, age at last contact, and alive versus deceased status as of last contact. Age at last contact was not a suitable approximation for our phenotype of interest, age at death, because 70% of our cohort was living. To produce a more homogeneous phenotype that would better estimate age at death, we calculated an expected age at death, which for deceased subjects was equal to their actual age at death, and for living subjects was equal to their age at last contact plus their age-specific and gender-specific life expectancies from life tables for the 1900 birth cohort [30]. The median year of birth for our cohort was 1901.

Since we could not predict what minimum age requirements would provide optimal power, ten sets of gender-specific minimum expected age at death requirements were applied to all subjects, with the hypothesis that as the cutoffs increased, the loss of power due to the decreasing number of families might be partially offset by an increase in genetic homogeneity and/or magnitude of effects in older subjects. As designated hereafter, Categories 1 to 10 cover the range from the upper 5% to the upper 0.2% tail of the birth cohort, corresponding to a minimum expected age at death of 90 to 100 for males and 95 to 104 for females (Table 1). Subjects that did not meet the age criteria for a category were removed from the analysis; for sibships with more than two siblings, individual siblings were excluded while retaining the sibship, whereas once either member of a sibling pair was eliminated, the entire sibship was removed.

**Table 1:** Subject characteristics by age category for Total group.

Age Category	1	2	3	4	5	6	7	8	9	10
**Upper tail of 1900 birth cohort**	5%	4%	3%	2.5%	2%	1.5%	1%	0.5%	0.3%	0.2%
**Minimum expected age at death (male)**	90	91	92	93	94	95	96	98	99	100
**Minimum expected age at death (female)**	95	96	97	98	99	100	101	102	103	104
**Sibships**	279	273	261	243	212	191	155	95	66	34
**2-ships**	218	217	209	209	185	172	140	86	61	32
**3-ships**	50	46	43	27	23	15	13	9	5	2
**4-ships**	9	8	7	5	4	4	2	0	0	0
**5-ships**	2	2	2	2	0	0	0	0	0	0
**% >2-ships**	21.9	20.5	19.9	14.0	12.7	9.9	9.7	9.5	7.6	5.9
**Average sibship size**	2.27	2.25	2.24	2.18	2.15	2.12	2.11	2.09	2.08	2.06
**Subjects**	632	614	585	529	455	405	327	199	137	70
**Average age at last contact**	99.4	99.5	99.7	100.0	100.4	100.6	101.0	101.8	102.2	103.1
**Subjects deceased at last contact**	192	186	174	161	136	114	92	60	33	15
**Subjects alive at last contact**	440	428	411	368	319	291	235	139	104	55
**Average life expectancy of subjects alive**	2.4	2.4	2.4	2.3	2.2	2.2	2.1	2.0	2.0	1.8
**Average expected age at death**	101.1	101.2	101.4	101.6	102.0	102.2	102.5	103.2	103.7	104.5
**Average expected age at death (male)**	99.3	99.4	99.5	99.5	99.9	99.9	100.2	101.2	101.6	102.6
**Average expected age at death (female)**	101.8	101.9	102.2	102.6	103.1	103.4	103.9	104.6	105.0	106.0
**Male subjects**	177	174	172	171	155	144	123	79	54	30
**Female subjects**	455	440	413	358	300	261	204	120	83	40
**% Male subjects**	28.0	28.3	29.4	32.3	34.1	35.6	37.6	39.7	39.4	42.9

Age, gender, and sibship composition statistics for the Total group are given across ten categories defined by gender-specific minimum requirements for expected age at death. The designations 2-ship, 3-ship, 4-ship, and 5-ship refer to sibships with two, three, four, or five siblings, respectively.

In addition to analyzing the complete Categories 1 to 10 (the “Total” group), we divided the sample set by two criteria into two subgroups for each. To explore differences between this study and the first scan [24], we analyzed (in Category 1) 129 of the 137 families used in the original cohort (the “Previous” subgroup) separately from 150 families recruited since that study was completed (the “New” subgroup). Because life expectancies show a gender bias, we also split the Total group into 140 sibships (in Category 1) with at least one male member (the male-containing, or “MC” subgroup) and 139 sibships comprised of only females (the female-only, or “FO” subgroup) (Table S1). Results that apply across all ten categories, in any group, are referred to as “Overall” for that group (for example, the Overall maximum LOD score).

### Genotyping and Filtering

Genomic DNA samples were purified from blood as described previously [24], and were subjected to whole genome amplification using the GenomiPhi kit (Amersham). Amplified DNA was genotyped at 10,204 single nucleotide polymorphisms (SNPs) on the GeneChip Human Mapping 10K 2.0 Array (Affymetrix). Samples that did not achieve at least a 95% SNP Call Rate were re-genotyped or excluded. Genotype concordance checks were performed to verify sibling status and eliminate duplicate samples, monozygotic twins, and unrelated subjects. Seven sibships were found to be comprised of at least one pair of half-siblings, which were included. Among 642 successfully genotyped samples, of which 632 subjects in 279 sibships qualified for Category 1, the mean SNP Call Rate was 98.81%, ranging from 95.03% to 99.87%. There were 109 SNPs that were not assigned to a chromosome, 170 SNPs that had a Hardy-Weinberg equilibrium *P* value below 10^−3^ among 282 unrelated subjects, and 267 SNPs that had a call rate of below 90% across all 642 samples. A total of 453 SNPs meeting one or more of these criteria was eliminated, resulting in a final panel of 9751 SNPs.

### Linkage Analysis

Linkage analysis was performed using MERLIN/MINX v1.1.2 [31]. Genotype data were converted into MERLIN-compatible input files using the Affymetrix tool GDASPort. All siblings were encoded as affected and their ungenotyped parents were encoded as phenotype unknown. Marker map positions based on the deCODE Genetics sex-averaged genetic map and marker allele frequencies for a Caucasian population were provided by Affymetrix. The Total group was also analyzed allowing MERLIN to calculate founder marker allele frequencies in each category separately, and the results were negligibly different than with the Affymetrix frequencies (data not shown).

We computed a multipoint nonparametric Kong and Cox allele-sharing LOD score with the S_all_ scoring function and the exponential allele-sharing model [32], since the commonly used nonparametric linkage (NPL) score is overly conservative with missing data [32]–[34] and our parental genotypes were universally absent. Hereafter, “LOD score” refers to these conditions. A parametric heterogeneity LOD (hLOD) score was also computed under both a dominant and recessive model, along with a conventional parametric LOD score. The penetrance for non-susceptible genotypes (phenocopy) under each model was arbitrarily set to one-tenth the birth cohort trait prevalence for that age category, and the penetrance for the susceptible genotypes was set to 1 since we analyzed only affected subjects. The “disease” allele frequencies were then calculated to fit the prevalence and penetrance model (Table S1). To limit the multiple testing burden, the results were screened only on the basis of the nonparametric LOD scores; parametric hLOD and LOD scores were noted only in genomic regions for which the nonparametric LOD scores were considered interesting (LOD≥2). The hLOD scores were used to verify that the nonparametric results were robust to different assumptions and provide information about the possible mode of inheritance of the putative longevity allele. Empirical *P* values were determined solely on the basis of nonparametric LOD scores.

We used the --rsq option in MERLIN [35] to accommodate linkage disequilibrium (LD) between markers. SNPs for which the pairwise coefficients of determination (r^2^) exceeded 0.16, above which LOD score inflation due to inter-marker LD becomes appreciable when parental genotypes are unobserved [36], were clustered with intervening SNPs and treated as a single multi-allelic marker. The error checking and Pedwipe functions of MERLIN were used to remove unlikely genotypes as implied by close double crossovers. Mendelian inheritance errors could not be identified due to the lack of parental genotypes.

### Empirical *P* Values

To account for the multiple partially-dependent hypotheses represented by the ten age categories, we calculated an Overall empirical *P* value for linkage peaks from any category in the Total group that met the significance threshold of LOD  = 3.6 for a single genomewide scan with a fully informative marker panel [37]. Based on the error-wiped pedigrees from Category 1, MERLIN was used to generate 1000 replicates of simulated genotype data under the null hypothesis of no linkage, applying the same marker map, allele frequencies, LD-corrected cluster definitions and haplotype frequencies (generated from the original scan with the --cfreq option), and missing data pattern as the actual data. The replicate pedigree files were successively trimmed nine times each to produce the corresponding pedigree files for Categories 2 to 10. These 1000 sets of ten replicates were analyzed identically to the actual data, and the number of distinct replicates or genomic locations in like replicates across all age categories that met or exceeded the observed LOD score for a given peak was recorded. If a replicate exceeded the observed LOD score in multiple categories at the same location, it was counted only once, just as in the original analysis.

We used the formula *P* = (*r*+1)/(*n*+1) to calculate empirical *P* values, where *r* is the number of replicates reaching the observed LOD score, and *n* is the number of replicates analyzed [38]. This formula produces a biased estimate of the true underlying *P* value, but provides a more accurate estimate of the type I error rate than the conventional, unbiased (yet anticonservative) formula *P* = *r*/*n* [38]–[41]. Upper 95% confidence limits for empirical *P* values were calculated using the conservative Clopper-Pearson exact method for a binomial distribution [42], as implemented in an online calculator [43].

## Results

**Figure 1:**
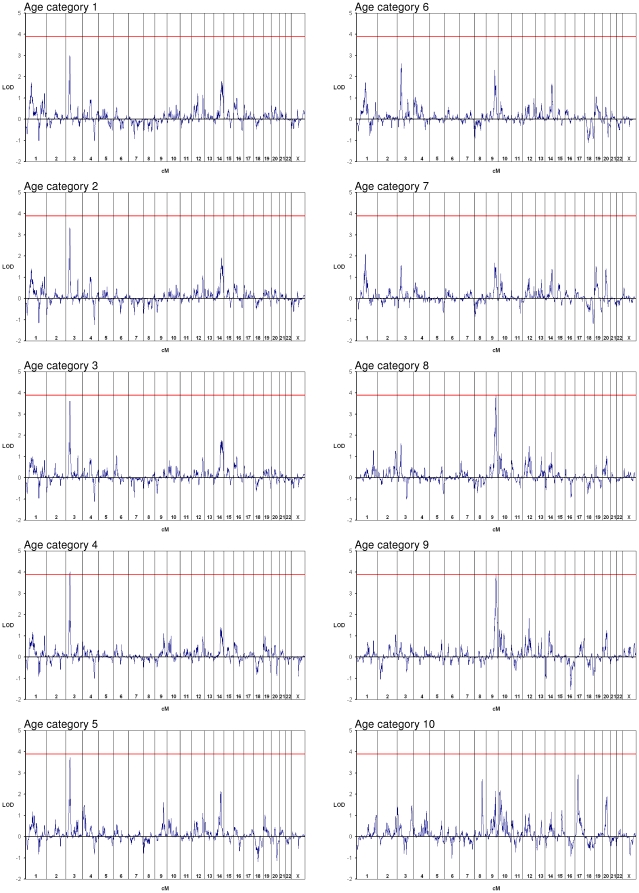
Genomewide nonparametric LOD scores by age category for Total group. Chromosome number is indicated at the bottom of each plot. Red line denotes empirical genomewide significance threshold across all age categories of LOD = 3.9.

In the Total group we identified a region on chromosome 3p24-22 with an Overall maximum LOD score of 4.02 in Category 4, and a region on chromosome 9q31-34 with an Overall maximum LOD score of 3.89 in Category 8. No other intervals achieved a LOD score above 3.0 (Figure 1), and given the high probability that peaks below that magnitude represent false positives, they were not considered further. By averaging the simulation results across all ten age categories, we calculated the per-category empirical threshold for genomewide significance with a type I error rate of α = 0.05 to be a LOD score of approximately 3.1, likely reflecting the imperfect informativity of the 10K panel [37]. Correcting for all categories, a LOD score of approximately 3.9 was required for Overall genomewide significance. For the chromosomes 3 and 9 linkage peaks, respectively, we observed 36 and 53 distinct genomic locations among 1000 sets of replicates for which a LOD score in any age category met or exceeded the observed scores, resulting in corresponding empirical *P* values of 0.037 [upper 95% confidence limit 0.048] and 0.054 [upper 95% confidence limit 0.067]. Only a single replicate achieved two LOD scores of at least 3.89 at distinct locations across all categories, yielding an empirical *P* value for the combined result of 0.002 [upper 95% confidence limit 0.006]. The stability of the chromosomes 3 and 9 linkage peaks was evaluated by dividing the marker panel into two independent subsets containing every other SNP, and both regions retained strong evidence for linkage in the halved panels (data not shown).

**Figure 2:**
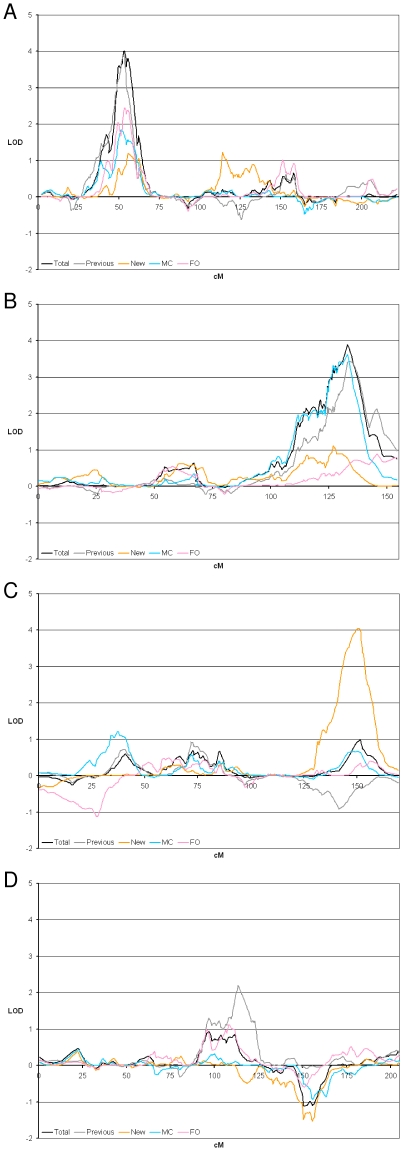
Nonparametric LOD scores by group for chromosomes 3, 9, 12, and 4 linkage peaks. LOD scores for the entire chromosome are plotted for the Category that produced the highest LOD score in the Total group for chromosomes 3 and 9, and the highest LOD score in the New and Previous subgroups for chromosomes 12 and 4, respectively. A) Chromosome 3, Category 4. B) Chromosome 9, Category 8. C) Chromosome 12, Category 6. D) Chromosome 4, Category 3.

The LOD scores for all linkage peaks varied considerably by group (Figure 2). The chromosome 4q22-25 linkage peak reported previously [24] produced an Overall maximum LOD score of 1.01 in Total Category 2. In the same region in the Previous subgroup we obtained maximum LOD scores of 2.14, 1.99, and 2.20 in Categories 1 to 3, respectively, whereas in the New subgroup, there was no evidence for linkage. For chromosomes 3 and 9, the Previous subgroup provided strong evidence for linkage with LOD scores of 3.90 in Category 4 (Overall maximum 4.49 in Category 5) and 3.43 in Category 8, respectively, while in the New subgroup the corresponding maximum LOD scores were 1.19 and 1.12. Analysis of the New subgroup also revealed a third novel locus on chromosome 12q24 with an Overall maximum LOD score of 4.05 in Category 6. This peak was completely absent from the Previous subgroup, and accordingly, the original cohort [24]. The Overall maximum LOD score for the chromosome 12 peak in the Total group was 1.11 in Category 5.

We did not discover any additional linkage peaks in the gender-stratified analyses. The chromosome 3 linkage peak was somewhat stronger in the FO group (Overall maximum LOD score of 2.77 in Category 5) than in the MC group (Overall maximum LOD score of 1.85 in Category 4), whereas the chromosome 9 linkage peak showed a greater difference in the opposite direction (Overall maximum LOD scores of 3.62 in MC Category 8 and 1.30 in FO Category 9, though the disparity may be explained by the greater number of MC than FO sibships in Categories 8 and 9). The subgroup characteristics are given in Table S1, and the complete nonparametric linkage data for the Total group and all four subgroups are provided in Text S1, S2, S3, S4 and S5.

The parametric analysis generally supported the results of the nonparametric analysis, showing all four linkage peaks in their respective groups and categories to be robust to different assumptions about the mode of inheritance. The chromosome 3 peak produced a higher maximum hLOD score under a recessive model (4.652) than a dominant model (3.334), whereas the maximum hLOD score for the chromosome 9 peak was higher under the dominant model (3.883) than the recessive model (2.680). The dominant model on chromosome 9 also yielded positive conventional parametric LOD scores of 1.047 in Category 8 and 2.840 in Category 9, suggesting reduced locus heterogeneity in the oldest sibships. The chromosome 12 peak achieved similar maximum hLOD scores of 3.915 and 3.875 under the dominant and recessive models, respectively, which likewise produced similar results on chromosome 4. We did not assess whether differences in hLOD scores under the dominant and recessive models were significant. Overall maximum nonparametric LOD and parametric hLOD scores, *P* values, and linkage peak locations for chromosomes 3, 9, 12, and 4 are given in Table 2.

**Table 2:** Characteristics of chromosomes 3, 9, 12, and 4 linkage peaks.

Locus	3p24.2–22.3	9q31.3–34.2	12q24.31–24.33	4q21.21–28.1
**Age category reported**	4	8	6	3
**Total max LOD (δ)**	4.02 (0.288)	3.89 (0.498)	1.11 (0.168)^a^	1.01 (0.135)^c^
**Total max hLOD dominant (α)**	3.334 (0.329)	3.883 (0.792)^b^	NA	NA
**Total max hLOD recessive (α)**	4.652 (0.271)	2.680 (0.364)^b^	NA	NA
**99% (−2 LOD) left confidence boundary**	rs2362772	rs723706	rs606443	rs726896
**Build 37.1 left boundary location**	24918232	112778426	120910630	81213792
**Peak maximum**	rs28150	rs536861	rs1732462	rs1008326
**Build 37.1 maximum location**	29594086	128313444	127444592	110563638
**99% (−2 LOD) right confidence boundary**	rs1382554	rs1074052^b^	rs953182	rs1586149
**Build 37.1 right boundary location**	35093841	136462498^b^	129106410	127074788
**Category-specific empirical ** ***P*** ** value**	0.006	0.012	NA	NA
**Overall empirical ** ***P*** ** value**	0.037	0.054	NA	NA
**Overall ** ***P*** ** value upper 95% confidence limit**	0.048	0.067	NA	NA
**Previous max LOD (δ)**	4.49 (0.464)^a^	3.43 (0.636)	<1	2.20 (0.298)
**Previous max hLOD dominant (α)**	4.029 (0.534)^a^	3.187 (0.833)	NA	1.954 (0.358)
**Previous max hLOD recessive (α)**	4.527 (0.389)^a^	2.766 (0.410)	NA	1.966 (0.241)
**New max LOD (δ)**	1.19 (0.220)	1.12 (0.377)	4.05 (0.504)	<1
**New max hLOD dominant (α)**	NA	NA	3.915 (0.605)	NA
**New max hLOD recessive (α)**	NA	NA	3.875 (0.386)	NA
**MC max LOD (δ)**	1.85 (0.260)	3.62 (0.603)	<1	<1
**MC max hLOD dominant (α)**	NA	3.145 (0.678)	NA	NA
**MC max hLOD recessive (α)**	NA	3.121 (0.377)	NA	NA
**FO max LOD (δ)**	2.77 (0.402)^a^	1.33 (0.622)^b^	<1	<1
**FO max hLOD dominant (α)**	2.499 (0.458)^a^	NA	NA	NA
**FO max hLOD recessive (α)**	3.500 (0.357)^a^	NA	NA	NA

Overall maximum LOD scores were noted if greater than or equal to 1, and hLOD scores were noted for peaks with LOD scores greater than or equal to 2. The parameters α and δ represent the proportion of families that are linked (parametric analyses), and the magnitude of excess allele-sharing (nonparametric analyses), respectively. Boundaries are for the Total group for chromosomes 3 and 9, the New subgroup for chromosome 12, and the Previous subgroup for chromosome 4. The boundaries of the chromosome 9 peak are a composite of Categories 8 and 9, which had overlapping but slightly offset peaks of similar magnitude. NA, not applicable (scores were not noted or *P* values were not determined).

a Category 5.

b Category 9.

c Category 2.

## Discussion

We performed the second genomewide linkage scan on families of exceptionally long-lived siblings, with substantial improvements in power over the first scan [24]. A filtered panel of 9751 SNPs with an average minor allele frequency of 0.27, equivalent to approximately 4000 microsatellite markers [44], provided a ten-fold effective increase in marker density over the original scan. The resulting advantage in information content and power of similar SNP arrays over microsatellite panels has been demonstrated in theory [45] and in practice [46]. Our analysis of 279 families in Total Category 1 included 129 of the 137 sibships in the previous study plus an independent cohort of 150 sibships. For a set of 300 sibpairs the power to detect linkage is nearly 100% for an allele conferring a two-fold increased risk to siblings of affected individuals [47], [48]. If genes with such magnitudes of effect exist they may be the most relevant to the study of human aging, and our scan would be well-powered to detect them.

Across ten categories of minimum expected age at death requirements, we identified three novel loci of interest: an Overall genomewide significant peak on chromosome 3p24-22 (LOD = 4.02, *P* = 0.037), a highly suggestive peak on chromosome 9q31-34 (LOD = 3.89, *P* = 0.054), and a peak on chromosome 12q24 (LOD = 4.05) in the newly recruited subset of our subjects. These linkage peaks are preliminary results that warrant replication studies, as several factors may influence their significance (Text S6). None of our scans provided substantial evidence for linkage to *FOXO3* on chromosome 6, probably because of the relatively small effect sizes conferred by SNPs at that locus [13]–[18]. Consistent with previous studies [24], [49], the *APOE* gene on chromosome 19 was likewise not linked in any analysis, again possibly due to the low relative mortality risks of the ε2 and ε4 haplotypes [50]. In addition, the ε4 haplotype predisposes young individuals to elevated mortality rates and is thus depleted in the exceptionally old [11], but such negatively selected alleles do not result in significant excess allele sharing among long-lived siblings [49]. By contrast, the ε2 haplotype is enriched in the exceptionally old [11], but the apparent protective effect may be mediated by a heterozygote advantage mechanism [50]-[52]. The ε2 haplotype is then analogous to a rare dominant variant, for which the power of allele-sharing methods is also low [49], [53].

The only previous genomewide linkage scan for exceptional longevity used a minimum actual age at death or last contact of 98 for probands and 91 or 95 for additional male or female siblings, respectively [24], most closely matching our Categories 1 and 2. The previous study produced distinct linkage peaks at both the 3p24-22 and 9q31-34 loci reported here, but with relatively small LOD scores of about 0.8 and 1.0, respectively. Our corresponding Overall maximum LOD scores for the two peaks in the Previous subgroup were 4.49 and 3.43 in Categories 5 and 8, but the scores in Category 1 were only 3.22 and 0.18. The chromosome 12 linkage peak reported here only in the New subgroup was completely absent from the previous study. Therefore, compared to the previous scan the discovery of the three novel loci was probably most attributable to different factors in each case: the increased informativity of the 10K marker panel for chromosome 3, the use of a range of age cutoffs for chromosome 9, and the expansion of our sample set for chromosome 12. Other previous scans using age-related traits have provided some evidence for linkage to chromosome 3p [25], [27], but given the disparate phenotypes and coarse microsatellite maps in these studies, it is difficult to assess the relevance of their data to our results.

Future linkage studies may benefit in power from denser marker panels [54] and larger sample sizes, but detection of loci with small effects and gene identification require other methods [55]. An association study of genes under the previously reported chromosome 4q22-25 linkage peak [24] implicated a SNP in the promoter of *MTTP* (MIM 157147) as associated with longevity [28], but multiple attempts to replicate this result were unsuccessful [12], [26], [28], [56]-[58]. It has been suggested that these studies also cast doubt on the prior linkage evidence [59], when in fact the two issues are largely independent, and this study was the first adequately powered attempt to replicate the previous scan. Although we were unable to reproduce the chromosome 4 linkage in the Total group or independent New subgroup, the evidence was stronger when only sibships used in the original study were analyzed (Overall maximum LOD score of 2.20 in Previous Category 3), indicating heterogeneity between subgroups. Importantly, only 129 of the 137 original sibships were available for this study, which could help account for the failure of our LOD score in Previous Category 1 (2.14) to reach the LOD score of 3.65 reported previously [24]. However, the Maximum LOD Score (MLS) statistic [60], [61] with the possible triangle constraint [62], as implemented by GeneHunter [33] in the original report [24], was later documented to result in a slight anticonservative bias that was most pronounced in small sample sizes [34]. The Kong and Cox LOD score [32] used here was not subject to that bias [34], possibly indicating the LOD score from the previous scan was somewhat inflated relative to our scan.

Several candidate genes in the linkage peaks discussed here warrant mention. The gene *TOP2B* (MIM 126431) in the chromosome 3 linkage peak encodes an isozyme of topoisomerase II, which is potently inhibited by resveratrol and related compounds from grape cell culture [63]. Resveratrol can mimic the effects of caloric restriction, mitigate the symptoms of age-related diseases, and/or extend lifespan in a variety of model organisms, including mammals [64], [65]. Various human topoisomerase homologs have also been shown to regulate cellular senescence [66], [67], promote telomere stability [68], and interact with the RecQ helicases encoded by *WRN* (MIM 604611) [69] and *BLM* (MIM 604610) [70], in which mutations cause the progeroid disorders Werner syndrome [71] and Bloom syndrome [72], respectively. *DBC1* (MIM 602865) in the chromosome 9 linkage peak directly interacts with *SIRT1* (MIM 604479) and inhibits its activity [73]; *SIRT1* is activated by resveratrol [74] and has been implicated in several age-related phenotypes in mammals [75]. The sirtuin family members are key regulators of lifespan in yeast [76], worms [77], and flies [78] and mediate the effects of caloric restriction [79], [80], the only behavior known to increase lifespan in a wide variety of organisms, including mammals [81]. In response to nutrient withdrawal, *SIRT1* is stimulated by *FOXO3* [82], in which variants have been reproducibly associated with human longevity [13]–[18]. In addition, different SNPs in or near *TLR4* (MIM 603030) in the chromosome 9 linkage peak have been associated at least once, though often not reproducibly, with exceptional longevity in men [83], [84], a bone-related proxy for biological age [85], and various age-related diseases [86]. Also of note, mutations in *ANK2* (MIM 106410), in the previously reported chromosome 4 linkage peak, cause long-QT syndrome [87], and common variation in *ANK2* has been reported to regulate the QT interval [88]. QT interval prolongation is a risk factor for sudden cardiac death in healthy individuals [89] as well as those with ischemic heart disease [90] and chronic congestive heart failure [91], which are more common causes of death among centenarians than younger individuals [92]. Heterozygous *Ank2* ^+/−^ knockout mice displayed multiple signs of premature senescence and their lifespan was significantly reduced compared to wild-type littermates [93]. Finally, a nominally significant association was reported between a SNP in *ALPK1* (MIM 607347) and both age at death and morbidity-free status at age 65 [85]. Both *ANK2* and *ALPK1* are within the 99% confidence (-2 LOD) [94] interval for the original chromosome 4 linkage peak [24] but are outside the 85% confidence (-1 LOD) interval tested in the study that identified *MTTP* [28], raising the possibility that *ANK2*, *ALPK1*, or another gene besides *MTTP* could explain the previous linkage result. The positions of all the above genes within linkage peaks suggest they merit attention in future gene identification efforts.

The linkage scans reported here should contribute to the analysis of both linkage and association studies on exceptional human longevity, which are underway in a massive data set [95]. Since cross-sectional study designs for longevity are subject to unique methodological complications [11] and longitudinal cohort designs can be prohibitively expensive, case-control studies will benefit from direction or corroboration by linkage scans. For example, the extensive multiple testing problem encountered in genomewide association studies can be partially alleviated by a weighted Bonferroni correction or Bayesian analysis that employs linkage data [96]. Alternatively, our results could help inform deep resequencing efforts to identify rare variants that influence lifespan, a particularly suitable approach for genes in which multiple variants exert individual effects too weak to be detected by association methods. Discovery and confirmation of human longevity genes will provide insight into the biology of aging and the genetic basis for resistance to age-related disease.

## Supporting Information

Table S1 Subject characteristics by age category for four subgroups, plus parametric analysis settings. Age, gender, and sibship composition statistics for the Previous, New, MC, and FO subgroups, plus parametric linkage parameters for all groups, are given across ten categories defined by gender-specific minimum requirements for expected age at death. The designations 2-ship, 3-ship, 4-ship, and 5-ship refer to sibships with two, three, four, or five siblings, respectively.
(0.14 MB DOC)

Text S1 Nonparametric linkage data for Total group.
(10.21 MB TXT)

Text S2 Nonparametric linkage data for Previous subgroup.
(10.05 MB TXT)

Text S3 Nonparametric linkage data for New subgroup.
(9.74 MB TXT)

Text S4 Nonparametric linkage data for MC subgroup.
(10.03 MB TXT)

Text S5 Nonparametric linkage data for FO subgroup.
(9.75 MB TXT)

Text S6 Supplemental discussion.
(0.05 MB DOC)
