# Supplementary material for: High-Density Genomewide Linkage Analysis of Exceptional Human Longevity Identifies Multiple Novel Loci
Source: PLoS One. 2010 Aug 31;5(8):e12432. doi: 10.1371/journal.pone.0012432 (PMC2930849; doi:10.1371/journal.pone.0012432)
Supplement: Table S1 — Subject characteristics by age category for four subgroups, plus parametric analysis settings. Age, gender, and sibship composition statistics for the Previous, New, MC, and FO subgroups, plus parametric linkage parameters for all groups, are given across ten categories defined by gender-specific minimum requirements for expected age at death. The designations 2-ship, 3-ship, 4-ship, and 5-ship refer to sibships with two, three, four, or five siblings, respectively. (0.14 MB DOC) [file pone.0012432.s001.doc]

**Table S1. Subject characteristics by age category for four subgroups, plus parametric analysis settings.**

| **Age Category** | **1** | **2** | **3** | **4** | **5** | **6** | **7** | **8** | **9** | **10** |
| --- | --- | --- | --- | --- | --- | --- | --- | --- | --- | --- |
| **Upper tail of 1900 birth cohort** | 5% | 4% | 3% | 2.5% | 2% | 1.5% | 1% | 0.5% | 0.3% | 0.2% |
| **Minimum expected age at death (male)** | 90 | 91 | 92 | 93 | 94 | 95 | 96 | 98 | 99 | 100 |
| **Minimum expected age at death (female)** | 95 | 96 | 97 | 98 | 99 | 100 | 101 | 102 | 103 | 104 |
| **Previous sibships** | 129 | 128 | 125 | 122 | 109 | 99 | 85 | 56 | 42 | 21 |
| **Previous 2-ships** | 95 | 96 | 97 | 104 | 94 | 89 | 77 | 50 | 39 | 20 |
| **Previous 3-ships** | 28 | 26 | 23 | 13 | 12 | 7 | 7 | 6 | 3 | 1 |
| **Previous 4-ships** | 4 | 4 | 3 | 3 | 3 | 3 | 1 | 0 | 0 | 0 |
| **Previous 5-ships** | 2 | 2 | 2 | 2 | 0 | 0 | 0 | 0 | 0 | 0 |
| **Previous % >2-ships** | 26.4 | 25.0 | 22.4 | 14.8 | 13.8 | 10.1 | 9.4 | 10.7 | 7.1 | 4.8 |
| **Previous average sibship size** | 2.33 | 2.31 | 2.28 | 2.20 | 2.17 | 2.13 | 2.11 | 2.11 | 2.07 | 2.05 |
| **Previous subjects** | 300 | 296 | 285 | 269 | 236 | 211 | 179 | 118 | 87 | 43 |
| **Previous average age at last contact** | 100.1 | 100.2 | 100.3 | 100.6 | 101.0 | 101.1 | 101.5 | 102.2 | 102.4 | 103.2 |
| **Previous subjects deceased at last contact** | 134 | 132 | 123 | 115 | 97 | 81 | 67 | 43 | 25 | 10 |
| **Previous subjects alive at last contact** | 166 | 164 | 162 | 154 | 139 | 130 | 112 | 75 | 62 | 33 |
| **Previous average expected age at death** | 101.3 | 101.4 | 101.6 | 101.8 | 102.2 | 102.4 | 102.8 | 103.5 | 103.8 | 104.6 |
| **Previous male subjects** | 79 | 79 | 77 | 77 | 72 | 67 | 58 | 40 | 30 | 17 |
| **Previous female subjects** | 221 | 217 | 208 | 192 | 164 | 144 | 121 | 78 | 57 | 26 |
| **Previous % male subjects** | 26.3 | 26.7 | 27.0 | 28.6 | 30.5 | 31.8 | 32.4 | 33.9 | 34.5 | 39.5 |
| **New sibships** | 150 | 145 | 136 | 121 | 103 | 92 | 70 | 39 | 24 | 13 |
| **New 2-ships** | 123 | 121 | 112 | 105 | 91 | 83 | 63 | 36 | 22 | 12 |
| **New 3-ships** | 22 | 20 | 20 | 14 | 11 | 8 | 6 | 3 | 2 | 1 |
| **New 4-ships** | 5 | 4 | 4 | 2 | 1 | 1 | 1 | 0 | 0 | 0 |
| **New 5-ships** | 0 | 0 | 0 | 0 | 0 | 0 | 0 | 0 | 0 | 0 |
| **New % >2-ships** | 18.0 | 16.6 | 17.6 | 13.2 | 11.7 | 9.8 | 10.0 | 7.7 | 8.3 | 7.7 |
| **New average sibship size** | 2.21 | 2.19 | 2.21 | 2.15 | 2.13 | 2.11 | 2.11 | 2.08 | 2.08 | 2.08 |
| **New subjects** | 332 | 318 | 300 | 260 | 219 | 194 | 148 | 81 | 50 | 27 |
| **New average age at last contact** | 98.8 | 99.0 | 99.1 | 99.4 | 99.8 | 100.0 | 100.4 | 101.2 | 101.8 | 102.9 |
| **New subjects deceased at last contact** | 58 | 54 | 51 | 46 | 39 | 33 | 25 | 17 | 8 | 5 |
| **New subjects alive at last contact** | 274 | 264 | 249 | 214 | 180 | 161 | 123 | 64 | 42 | 22 |
| **New average expected age at death** | 100.9 | 101.0 | 101.1 | 101.4 | 101.7 | 101.9 | 102.2 | 102.8 | 103.5 | 104.3 |
| **New male subjects** | 98 | 95 | 95 | 94 | 83 | 77 | 65 | 39 | 24 | 13 |
| **New female subjects** | 234 | 223 | 205 | 166 | 136 | 117 | 83 | 42 | 26 | 14 |
| **New % male subjects** | 29.5 | 29.9 | 31.7 | 36.2 | 37.9 | 39.7 | 43.9 | 48.1 | 48.0 | 48.1 |
| **MC sibships** | 140 | 137 | 136 | 135 | 122 | 113 | 94 | 61 | 43 | 22 |
| **MC 2-ships** | 97 | 97 | 99 | 110 | 103 | 100 | 82 | 53 | 38 | 20 |
| **MC 3-ships** | 32 | 30 | 28 | 18 | 15 | 9 | 10 | 8 | 5 | 2 |
| **MC 4-ships** | 9 | 8 | 7 | 5 | 4 | 4 | 2 | 0 | 0 | 0 |
| **MC 5-ships** | 2 | 2 | 2 | 2 | 0 | 0 | 0 | 0 | 0 | 0 |
| **MC % >2-ships** | 30.7 | 29.2 | 27.2 | 18.5 | 15.6 | 11.5 | 12.8 | 13.1 | 11.6 | 9.1 |
| **MC average sibship size** | 2.40 | 2.38 | 2.35 | 2.25 | 2.19 | 2.15 | 2.15 | 2.13 | 2.12 | 2.09 |
| **MC subjects** | 336 | 326 | 320 | 304 | 267 | 243 | 202 | 130 | 91 | 46 |
| **% MC sibships in Total** | 50.2 | 50.2 | 52.1 | 55.6 | 57.5 | 59.2 | 60.6 | 64.2 | 65.2 | 64.7 |
| **Males in MC sibships** | 177 | 174 | 172 | 171 | 155 | 144 | 123 | 79 | 54 | 30 |
| **Females in MC sibships** | 159 | 152 | 148 | 133 | 112 | 99 | 79 | 51 | 37 | 16 |
| **% Males in MC sibships** | 52.7 | 53.4 | 53.8 | 56.3 | 58.1 | 59.3 | 60.9 | 60.8 | 59.3 | 65.2 |
| **FO sibships** | 139 | 136 | 125 | 108 | 90 | 78 | 61 | 34 | 23 | 12 |
| **FO 2-ships** | 121 | 120 | 110 | 99 | 82 | 72 | 58 | 33 | 23 | 12 |
| **FO 3-ships** | 18 | 16 | 15 | 9 | 8 | 6 | 3 | 1 | 0 | 0 |
| **FO 4-ships** | 0 | 0 | 0 | 0 | 0 | 0 | 0 | 0 | 0 | 0 |
| **FO 5-ships** | 0 | 0 | 0 | 0 | 0 | 0 | 0 | 0 | 0 | 0 |
| **FO % >2-ships** | 12.9 | 11.8 | 12.0 | 8.3 | 8.9 | 7.7 | 4.9 | 2.9 | 0.0 | 0.0 |
| **FO average sibship size** | 2.13 | 2.12 | 2.12 | 2.08 | 2.09 | 2.08 | 2.05 | 2.03 | 2.00 | 2.00 |
| **FO subjects** | 296 | 288 | 265 | 225 | 188 | 162 | 125 | 69 | 46 | 24 |
| **Phenocopy rate** | 0.0050 | 0.0040 | 0.0030 | 0.0025 | 0.0020 | 0.0015 | 0.0010 | 0.0005 | 0.0003 | 0.0002 |
| **Dominant disease allele frequency** | 0.0229 | 0.0182 | 0.0136 | 0.0113 | 0.0091 | 0.0068 | 0.0045 | 0.0023 | 0.0014 | 0.0009 |
| **Recessive disease allele frequency** | 0.2127 | 0.1901 | 0.1646 | 0.1502 | 0.1343 | 0.1163 | 0.0949 | 0.0671 | 0.0520 | 0.0424 |

Age, gender, and sibship composition statistics for the Previous, New, MC, and FO subgroups, plus parametric linkage parameters for all groups, are given across ten categories defined by gender-specific minimum requirements for expected age at death. The designations 2-ship, 3-ship, 4-ship, and 5-ship refer to sibships with two, three, four, or five siblings, respectively.
