# Supplementary material for: High-Density Genomewide Linkage Analysis of Exceptional Human Longevity Identifies Multiple Novel Loci
Source: PLoS One. 2010 Aug 31;5(8):e12432. doi: 10.1371/journal.pone.0012432 (PMC2930849; doi:10.1371/journal.pone.0012432)
Supplement: Text S6 — Supplemental discussion. (0.05 MB DOC) [file pone.0012432.s007.doc]

**Text S6. Supplemental discussion.**

Several features of our study design and results may affect the interpretation of the linkage peaks reported herein. First, the genomewide average LOD scores in Total Categories 1 to 10 ranged from 0.077 to 0.129, indicating a slight excess of allele-sharing compared to the expected average LOD score of 0 under the null hypothesis of no linkage. The lowest Overall LOD score in the Total group was ‑1.54 in Category 9; the scarcity and low absolute values of negative LOD scores (Figure 1) suggest there were no major problems with genotyping accuracy or the pedigree structures. Still, the data cleaning and error checking we performed could not have removed every error from the data set, and we observed a consistent tendency of the error checking function in MERLIN to increase LOD scores in linkage peaks. Therefore we would expect our imperfectly cleaned data to generate slightly lower LOD scores than the error-free simulated data, so the empirical *P* values are likely somewhat conservative. In addition, the Clopper-Pearson exact method we used to calculate confidence intervals provides an actual coverage probability guaranteed to be greater than or equal to the nominal coverage probability, resulting in a conservative interval [1]. We also used approximate methods that are less conservative, and as expected, the resulting intervals had slightly lower upper limits but were close to the exact intervals due to our *n* of 1000 (data not shown).

Our phenotype of expected age at death applied an age‑specific and gender-specific U.S. population average for additional life expectancy to living individuals, similar to a recently reported method [2], meaning that the life expectancy for a subject of age A was conditional on having reached age A. However, because our subjects had long-lived siblings they were likely more enriched for longevity alleles than the general population of long-lived individuals. Life tables would then underestimate their life expectancies, making our approach conservative. It is therefore possible that an alternative method, such as the Family Longevity Selection Score [2] would be more powerful. However, due to the high mortality rates among the very old, the average additional life expectancy for the living subjects in Total Category 1 was only 2.4 years. Hence it is unlikely that a different method for incorporating alive versus deceased status or simply using age at last contact would have produced substantially different results.

The two sets of two subgroups formed by splitting our Total group represent four additional, partially-dependent hypotheses. Although we screened the results from these four subgroups genomewide in all age categories, to reduce the computational burden of the simulations we did not evaluate the significance of linkage peaks obtained in the subgroups. It is then unclear to what extent the testing of subgroups would affect the significance of the Total results on chromosomes 3 and 9. Given the empirical *P* value of 0.002 for the combined result it is unlikely that two such loci would have arisen by chance under the null hypothesis of no linkage, but both peaks individually require replication.

All four linkage peaks discussed herein displayed a difference greater than two in their Overall maximum LOD scores between the subgroups defined by time of enrollment. Compared to the New subgroup, the Previous subgroup had several differences in composition that might explain its relatively high contribution to the chromosomes 3, 9, and 4 linkage peaks, including a greater number of sibships in Categories 5 to 10, a slight enrichment for larger sibships in Categories 1 to 5, a lower percentage of male subjects, and an average 0.5-year higher average expected age at death across all categories (Table S1). The median years of birth for the Previous and New subgroups were 1900 and 1903, respectively, so it is also conceivable that shifting demographics resulted in dissimilar ancestry between subgroups. Other likely explanations for discrepancies in LOD scores include locus heterogeneity [3], by which a random splitting of the samples could enrich or deplete a particular subgroup for certain alleles, and stochastic effects [4], since both chromosomes 12 and 4 showed a negative LOD score trough (for which there is no biological reason in nonparametric linkage analysis) in either the Previous or New subgroup that corresponded to a positive peak in the other subgroup. These hypotheses are consistent with the large variances in magnitude and position of linkage peaks generated from fewer than 500 sibpairs [4].

Different linkage peaks were most pronounced in certain age categories and diminished in others. Loss of power due to decreased sample size was expected to decrease LOD scores in higher categories, while the older ages of subjects and the corresponding increase in the magnitude of genetic effect sizes were expected to increase LOD scores in higher categories. The balance of these two opposing forces for any particular linkage peak, in combination with stochastic effects, likely determined the distribution of its LOD scores across all ten categories. For example, while the chromosome 3 peak was somewhat robust to age category in that its LOD score was greater than 2 in Categories 1 to 6, it was reduced in the highest categories. It is possible that for some intervals any gains in power due to an enrichment of genetic factors in higher age categories were outweighed by the corresponding loss of power due to smaller sample sizes. Each incremental increase of one year in the minimum expected age at death resulted in a substantial increase in the proportion of families lost from the previous category, ranging from 2% fewer families in Category 2 than 1, to 48% fewer in Category 10 than 9. By contrast, the chromosome 9 peak was most evident in Categories 8 and 9, consistent with an age-dependent effect, a loss of power in lower categories due to heterogeneity and in Category 10 due to sample size, or a false positive result. Recently developed methods for selecting and weighting sibships in linkage scans [2,5-7] might eliminate the need to analyze age categories, thereby reducing multiple hypothesis testing and taking better advantage of sibships with large differences in age between siblings. While future studies will likely benefit in power from these weighting approaches, it is possible they may fail to detect a linkage peak such as our peak on chromosome 9 that arose through the complete removal of younger subjects.

Comparing other aspects of the reported linkage peaks, the maximum LOD score for the chromosome 9 peak was slightly lower than for chromosome 3, but the magnitude of the genetic effect, as estimated by the parameter δ [8], was higher for chromosome 9 (0.498 in Category 8 and 0.620 in Category 9, versus 0.288 in Category 4 and 0.298 in Category 5 for chromosome 3). This observation corresponds to the chromosome 9 peak reaching a comparable LOD score to the chromosome 3 peak in a sample set of many fewer families, and supports the hypothesis that genetic effect sizes may have been largest in the highest age categories. The chromosome 12 peak in New Category 6 was similar to the chromosome 9 peak in Total Category 8 in maximum LOD score, number of families, and δ value, possibly suggesting a relatively large effect size for the putative chromosome 12 gene as well. Finally, chromosome 9 yielded an Overall maximum conventional parametric LOD score in Category 9 of 2.840 under a dominant model, indicating that locus heterogeneity was sufficiently low in the oldest sibships that summing the per-family parametric LOD scores across all families produced a suggestive result.

**References**

1. Brown LD, Cai TT, DasGupta A (2001) Interval estimation for a binomial proportion. Statistical Science 16: 101-133.

2. Sebastiani P, Hadley EC, Province M, Christensen K, Rossi W, et al. (2009) A family longevity selection score: ranking sibships by their longevity, size, and availability for study. Am J Epidemiol 170: 1555-1562.

3. Gao X, Martin ER, Liu Y, Mayhew G, Vance JM, et al. (2009) Genome-wide linkage screen in familial Parkinson disease identifies loci on chromosomes 3 and 18. Am J Hum Genet 84: 499-504.

4. Cordell HJ (2001) Sample size requirements to control for stochastic variation in magnitude and location of allele-sharing linkage statistics in affected sibling pairs. Ann Hum Genet 65: 491-502.

5. Houwing-Duistermaat JJ, Callegaro A, Beekman M, Westendorp RG, Slagboom PE, et al. (2009) Weighted statistics for aggregation and linkage analysis of human longevity in selected families: the Leiden Longevity Study. Stat Med 28: 140-151.

6. Callegaro A, van Houwelingen HC, Houwing-Duistermaat JJ (2009) Score test for age at onset genetic linkage analysis in selected sibling pairs. Stat Med 28: 1913-1926.

7. Callegaro A, Uh HW, Helmer Q, Houwing-Duistermaat JJ (2009) New score tests for age-at-onset linkage analysis in general pedigrees. BMC Proc 3 Suppl 7: S97.

8. Kong A, Cox NJ (1997) Allele-sharing models: LOD scores and accurate linkage tests. Am J Hum Genet 61: 1179-1188.
